# Lobectomy for lung cancer with a displaced left B^1 + 2^ and an anomalous pulmonary vein: a case report

**DOI:** 10.1186/s13019-021-01392-3

**Published:** 2021-01-21

**Authors:** Shinichi Sakamoto, Hiromitsu Takizawa, Naoya Kawakita, Akira Tangoku

**Affiliations:** Department of Thoracic, and Endocrine Surgery and Oncology, Institute of Biomedical Sciences, The University of Tokushima Graduate School, 3-18-15, Kuramoto-cho, Tokushima, 770-8503 Japan

**Keywords:** Displaced bronchus, Anomalous pulmonary vein, Lung cancer

## Abstract

**Background:**

A displaced left B^1 + 2^ accompanied by an anomalous pulmonary vein is a rare condition involving complex structures. There is a risk of unexpected injuries to bronchi and blood vessels when patients with such anomalies undergo surgery for lung cancer.

**Case presentation:**

A 59-year-old male with suspected lung cancer in the left lower lobe was scheduled to undergo surgery. Chest computed tomography revealed a displaced B^1 + 2^ and hyperlobulation between S^1 + 2^ and S^3^, while the interlobar fissure between S^1 + 2^ and S^6^ was completely fused. Three-dimensional computed tomography (3D-CT) revealed an anomalous V^1 + 2^ joining the left inferior pulmonary vein and a branch of the V^1 + 2^ running between S^1 + 2^ and S^6^. We performed left lower lobectomy via video-assisted thoracic surgery, while taking care with the abovementioned anatomical structures. The strategy employed in this operation was to preserve V^1 + 2^ and confirm the locations of B^1 + 2^ and B^6^ when dividing the fissure.

**Conclusion:**

The aim of the surgical procedure performed in this case was to divide the fissure between S^1 + 2^ and the inferior lobe to reduce the risk of an unexpected bronchial injury. 3D-CT helps surgeons to understand the stereoscopic positional relationships among anatomical structures.

## Background

Due to the development and spread of imaging technology, thoracic surgeons are able to obtain a precise understanding of the anatomical structures of patients’ lungs before surgery [1]. Surgeons should plan surgical procedures using imaging modalities to avoid unexpected injuries to anatomical structures because the pulmonary anatomy exhibits numerous variations and anomalies. Herein, we report an exceedingly rare case involving a patient with a displaced B^1 + 2^ accompanied by an anomalous V^1 + 2^, which joined the left inferior pulmonary vein. We were able to safely perform left lower lobectomy using video-assisted thoracic surgery (VATS) after preoperatively identifying these complex anatomical structures.

## Case presentation

**Fig. 1:**
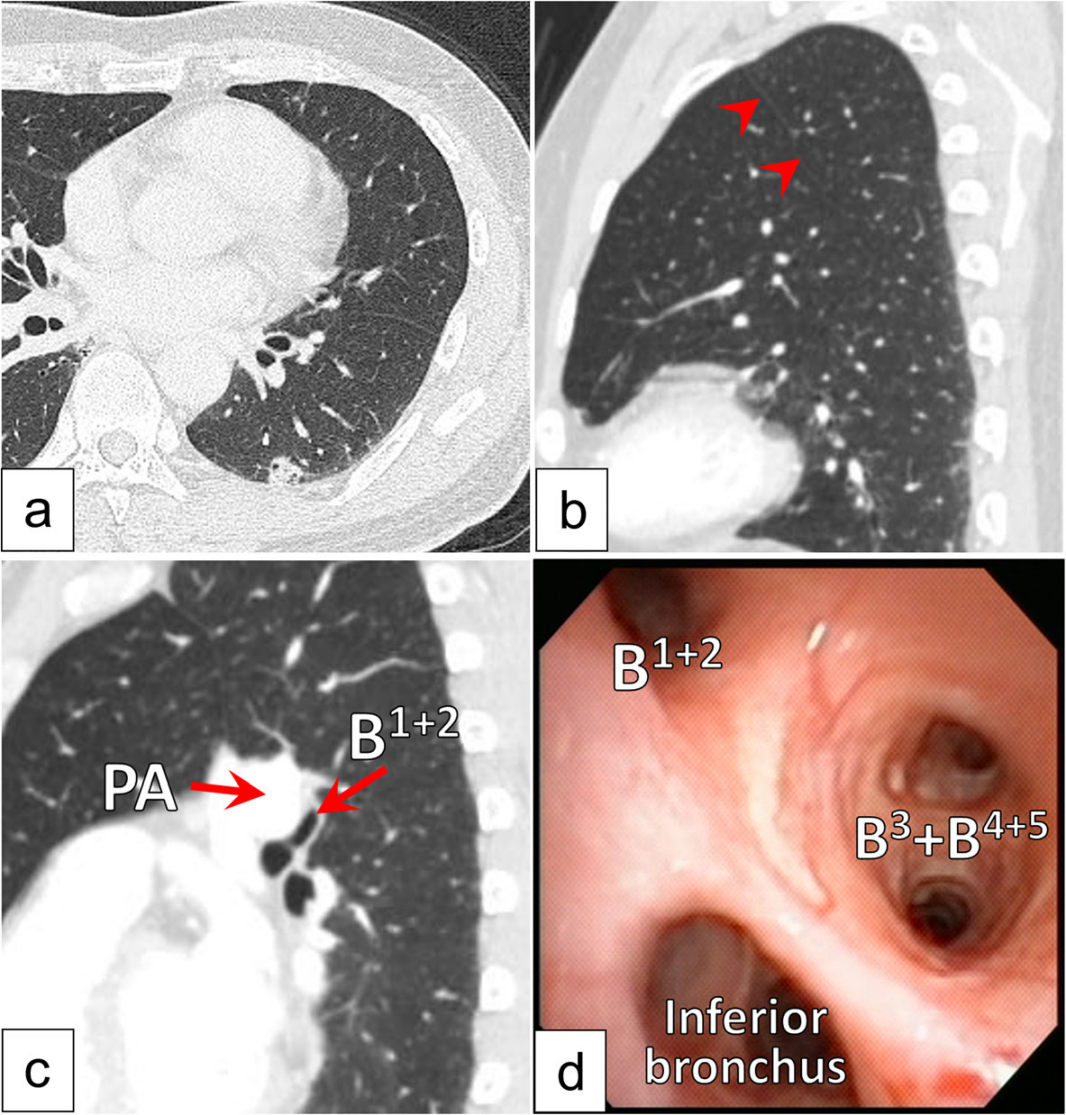
Preoperative chest computed tomography images and bronchoscopic image. Chest computed tomography (CT) revealed a tumor in the left inferior lobe (**a**) and hyperlobulation between S^1 + 2^ and S^3^ (red arrow), while the interlobar fissure between S^1 + 2^ and S^6^ was completely fused (**b**). CT revealed a displaced B^1 + 2^ running behind the main pulmonary artery (**c**). Bronchoscopy revealed that three bronchi branched from the left main bronchus (**d**).

**Fig. 2:**
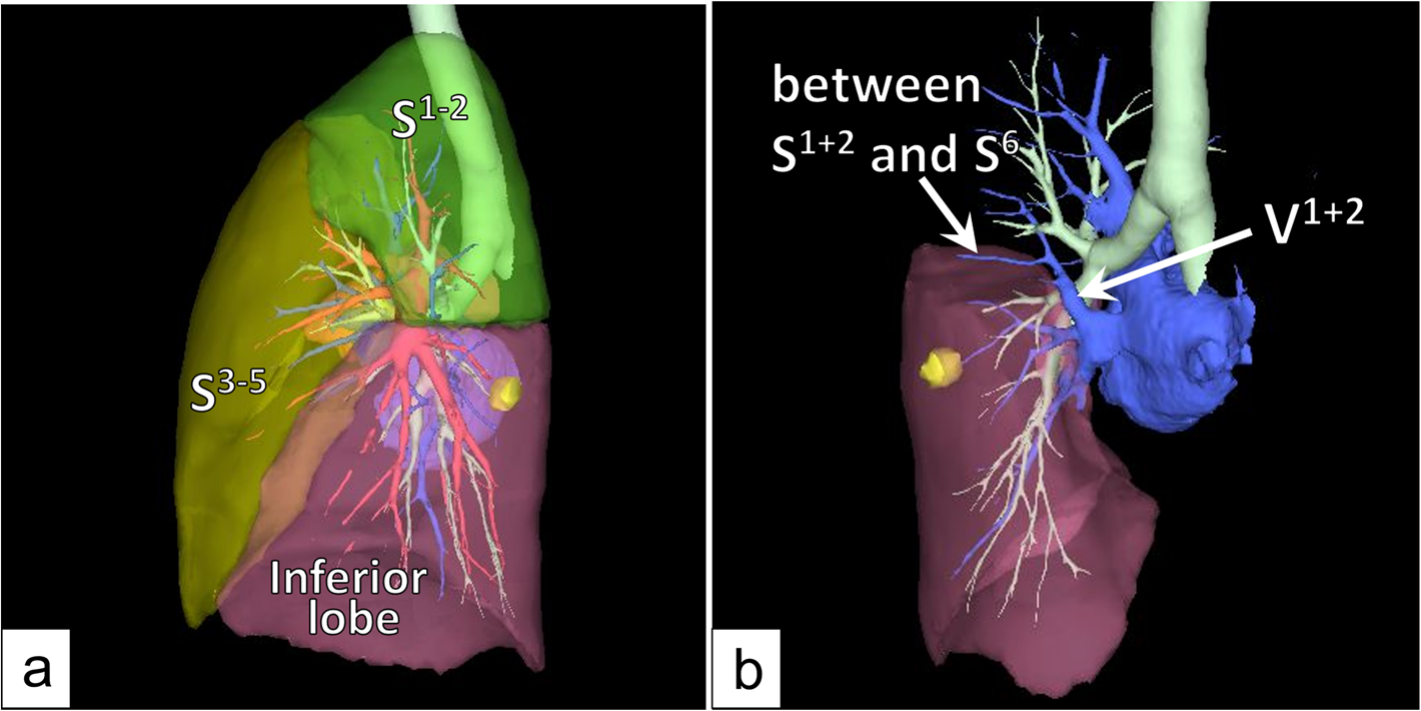
Preoperative three-dimensional chest computed tomography images. 3D-CT revealed that the volume of S^1 + 2^ was relatively large (**a**). The anomalous V^1 + 2^ ran from S^1 + 2^ to the left inferior pulmonary vein, and a branch of V^1 + 2^ ran between S^1 + 2^ and S^6^ (**a**, **b**).

**Fig. 3:**
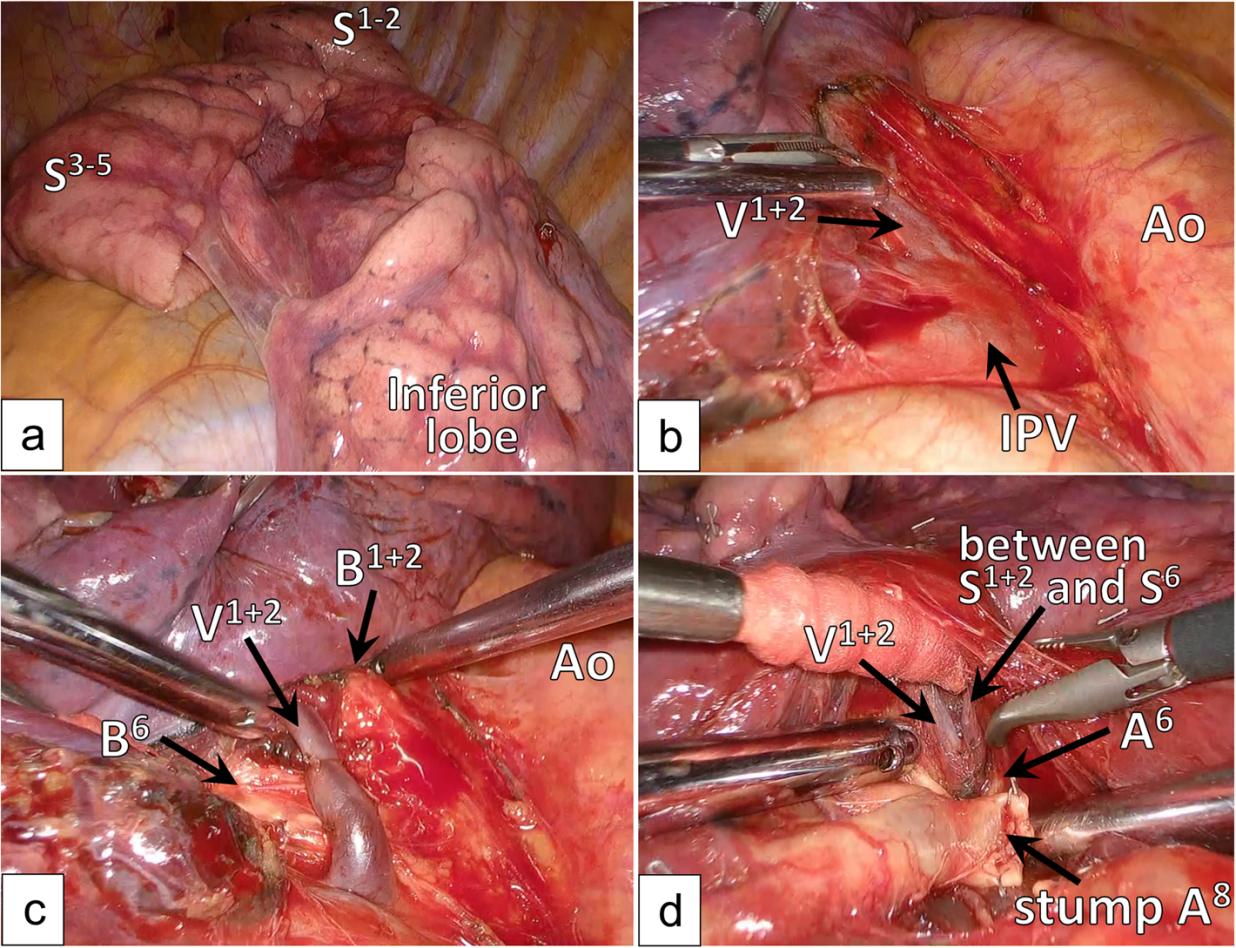
Intraoperative findings. Hyperlobulation was seen between S^1 + 2^ and S^3^, and a largely fused fissure was observed between S^1 + 2^ and S^6^ (**a**). The inferior pulmonary vein (IPV) was identified on the posterior side of the mediastinal pleura (**b**). In the process of peeling away the proximal V^1 + 2^, we were able to clearly distinguish between B^1 + 2^ and B^6^ on the posterior side (**c**). The branch of V^1 + 2^ running between S^1 + 2^ and S^6^ was used as a landmark when dividing the fissure between S^1 + 2^ and the inferior lobe (**d**)

A 59-year-old male with no history of smoking exhibited a slightly increased carcinoembryonic antigen level (5.6 ng/mL) during a health check. Chest computed tomography (CT) revealed a tumor (maximum diameter: 13 mm) in the left lower pulmonary lobe (Fig. 1a). He was referred to our hospital with suspected left lower lobe lung cancer (cT1bN0M0 stage1A2). CT and three-dimensional CT (3D-CT), which was performed using the Fujifilm Synapse Vincent system (Fujifilm Corporation, Tokyo, Japan), revealed the following anatomical anomalies in the left lung: 1) a displaced B^1 + 2^ running behind the main pulmonary artery, 2) an anomalous V^1 + 2^ joining the left inferior pulmonary vein (Fig. 2b), and 3) hyperlobulation between S^1 + 2^ and S^3^ with a completely fused interlobar fissure between S^1 + 2^ and S^6^ (Fig. 1b and c). 3D-CT also indicated that the interlobar plane between S^1 + 2^ and S^6^ ran perpendicular to the cranio-caudal direction because the volume of S^1 + 2^ was relatively large (Fig. 2a). Bronchoscopy revealed that three bronchi branched from the left main bronchus (Fig. 1d).

We planned VATS for surgical diagnosis and treatment. Hyperlobulation between S^1 + 2^ and S^3^ and a fused fissure between S^1 + 2^ and S^6^ were observed (Fig. 3a). At first, we performed non-anatomical wedge resection of the lesion to achieve a rapid pathological diagnosis. The patient was diagnosed with adenocarcinoma, and left lower lobectomy and systematic nodal dissection were performed.

The major pitfalls that we had to pay attention to during this surgery were as follows: 1) to avoid injuring the displaced B^1 + 2^ running behind the main pulmonary artery and 2) to avoid cutting the anomalous V^1 + 2^.

The inferior pulmonary vein was identified on the posterior side of the hilum, and the anomalous V^1 + 2^ joined it (Fig. 3b). To prevent B^1 + 2^ from being mistaken for B^6^, we distinguished B^1 + 2^ from the distal section of B^6^ on the posterior side (Fig. 3c). The distal branch of A^8^ was identified using the interlobar fissure. After A^8^ was divided, we peeled away the pulmonary artery in the proximal direction to identify A^6^ and V^1 + 2^, which ran near A,^6^ and a branch of V^1 + 2^, which ran between S^1 + 2^ and S^6^ (Figs. 2b and 3d). This branch was used as a landmark when we divided the fissure between S^1 + 2^ and the inferior lobe. Forceps were passed from the anterior to posterior side between a branch of V^1 + 2^ and A,^6^ and the largely fused fissure between S^1 + 2^ and the inferior lobe was divided using a stapler. After dividing the fissure, A^6^ and A^9 + 10^ were identified and divided. The inferior bronchus branched from the left main bronchus at the level of the branches of B^3^ + B^4 + 5^ and the displaced B^1 + 2^, which was located at a more proximal site than normal; therefore, we needed to peel away the bronchus while holding down the pulmonary artery and identified the station 11 lymph nodes. Forceps were passed from the anterior to the posterior side along the station 11 lymph nodes, and the incomplete fissure between S^5^ and inferior lobe was divided using the stapler. After dividing the fissure, the inferior bronchus was divided, which completed the lobectomy ND2a-2 procedure.

The operation time was 185 min, and 30 mL intraoperative blood loss occurred. Pathologically, the tumor was diagnosed as an invasive mucinous adenocarcinoma with a maximal diameter of 15 mm, and the pathological stage was p-T1aN0M0 stage I A1. The patient’s postoperative course was uneventful, and he was discharged from hospital 6 days after the surgery.

## Discussion and conclusion

In lung surgery, numerous variations in anatomical structures can be encountered, and they can cause serious complications if surgeons do not identify them preoperatively. Foster-Carter reported that bronchial anomalies are present in 0.64% of patients and classified them into displaced bronchi; i.e., bronchi that branch away from their normal positions, and supernumerary bronchi; i.e., excess bronchi that occur in addition to the normal bronchi [2]. Eighty percent of displaced bronchi occur in the right upper lobe, and 11% are displaced B^1 + 2^ arising from the left main bronchus [3]. In our case, a displaced left B^1 + 2^ accompanied by an anomalous V^1 + 2^ of the left pulmonary vein was seen, which is even rarer than the abovementioned variation. In addition, a case involving a displaced left B^1 + 2^, in which there were accessory fissures between the segment containing the displaced bronchus and an incomplete fissure between the upper lobe and lower lobe, was reported [4]. Thus, in the present case we needed to consider how we should create a fissure. Three surgical cases involving displaced B^1 + 2^, in which unexpected B^1 + 2^ injuries occurred when a region of lobulation between S^1 + 2^ and S6 was divided, even though two cases were recognized anomalies before surgery, have been reported (Table 1) [7–9]. The injuries were caused by the displaced B^1 + 2^ being misidentified as B^6^ when the fissure between S^1 + 2^ and the inferior lobe was divided using a stapler. Since excision of the upper lobe was scheduled in both cases, the injuries did not have adverse effects on the surgical procedures. However, unexpected injuries to the upper lobe can be serious complications during lower lobectomy. The aim of the surgical procedure performed in the present case was to divide the fissure between S^1 + 2^ and the lower lobe. It was necessary to take great care during this procedure because there was an anomalous V^1 + 2^ that passed from S^1 + 2^ to the inferior pulmonary vein in this case. In such cases, it is important to be aware not only of the existence of the anomaly, but also to understand the stereoscopic positional relationships between the pulmonary veins and bronchi. In the present case, 3D-CT revealed a displaced B^1 + 2^ arising from the left main bronchus and running along the left edge of the main pulmonary artery. In addition, an anomalous V^1 + 2^ was found to run between B^1 + 2^ and B^6^ and the dorsal side of the inferior bronchus. The branch of V^1 + 2^ running between S^1 + 2^ and S^6^ was used as a landmark when dividing the fissure between S^1 + 2^ and S^6^. By peeling away the distal part of V^1 + 2^ using a posterior approach, we were able to clearly identify B^6^, B^1 + 2^, and A.^6^ These anatomical findings matched the preoperative 3D-CT findings [1].

**Table 1:** Previous reports of lung resection in patients with a displaced left B^1+ 2^

Author /year	Age /sex	Diagnosis	Displaced Br	Procedure	Anomalous PV	Fissure	Injury	Cause of injury	Preoperative recognition of anomalies
Shimamoto /2008 [5]	81/F	Carcinoma	B^1 + 2^	S^1 + 2^ Seg.	–	S^1–5^/ S^6–10^	B^1 + 2^	Dividing fissure between S^1 + 2^ and S^6^	+
Tsukioka /2011 [6]	62/F	Carcinoma	B^1 + 2^	LUL	–	S^1, 2^/ S^3–10^	B^1 + 2^	Dividing fissure between S^1 + 2^ and S^6^	+
Osawa /2018 [7]	57/F	Carcinoma	B^1 + 2^	S^1 + 2^ Seg.	–	S^1 + 2,6–10^/ S^3–5^	B^1 + 2^	Dividing fissure between S^1 + 2^ and S^6^	–

*Br* Bronchus, *PV* Pulmonary vein, *F* Female, *Seg*. Segmentectomy, *LUL* Left upper lobectomy

In this case, 3D-CT helped us to share information across the surgical team, which allowed the surgeons to become familiar with the patient’s rare anatomical structures. It is imperative for surgeons to acquire complete information about patients’ pulmonary anatomies before surgery, including a full understanding of any rare anomalies or complicated anatomies, before performing lobectomy.

In conclusion, during left lower lobectomy for patients with displaced B^1 + 2^, dividing the fissure between S^1 + 2^ and S^6^ carries a risk of injuring a bronchus. 3D-CT helped us to obtain information about the patient’s anatomy, leading to an optimal preoperative assessment and appropriate strategic planning.

## Data Availability

Not applicable.

## Abbreviations

CT: Computed tomography
3D-CT: Three-dimensional computed tomography
VATS: Video-assisted thoracic surgery
